# Association of Gut Microbiota-Derived Short-Chain Fatty Acids With Persistent Elevated Serum Transaminase Levels in Normal Weight and Obesity: A Pilot Study

**DOI:** 10.1155/jnme/6652392

**Published:** 2025-09-20

**Authors:** David Alberto Díaz de Sandy-Galán, Hugo Villamil-Ramírez, Maricela Rodríguez-Cruz, Blanca López-Contreras, Paola León-Mimila, Marisol Olivares-Arévalo, Jorge Maldonado-Hernández, Israel Domínguez-Calderon, Jorge Salmerón, Daniel Cerqueda-García, Teresa Villarreal-Molina, Rafael Velázquez-Cruz, Samuel Canizales-Quinteros

**Affiliations:** ^1^Unit of Population Genomics Applied to Health, Department of Biology, School of Chemistry, National Autonomous University of Mexico (UNAM), National Institute of Genomic Medicine (INMEGEN), Mexico City 14610, Mexico; ^2^Laboratory of Molecular Nutrition, Medical Nutrition Research Unit, Pediatric Hospital, National Medical Center “21st Century”, Mexican Social Security Institute (IMSS), Mexico City 06720, Mexico; ^3^Research Center in Policies, Population and Health, School of Medicine, UNAM, Mexico City 04510, Mexico; ^4^Biorational Pest and Vector Management Network, Biomimic® Scientific and Technological Cluster, Ecology Institute AC, Xalapa 91073, Mexico; ^5^Genomics of Cardiovascular Disease Laboratory, INMEGEN, Mexico City 14610, Mexico; ^6^Genomics of Bone Metabolism Laboratory, INMEGEN, Mexico City 14610, Mexico

**Keywords:** gut microbiota, hepatic steatosis, short-chain fatty acids, transaminases

## Abstract

Although obesity is the most common risk factor for hepatic steatosis, this disease may occur in normal-weight individuals. While gut microbial metabolites such as short-chain fatty acids (SCFAs) have been associated with obesity and metabolic disease, the relationship among fecal SCFA concentrations, SCFA-producing bacteria, and hepatic steatosis with and without obesity is not fully understood. This pilot study aimed to compare fecal SCFA concentrations and SCFA-producing gut bacteria in four study groups: 7 individuals with normal-weight and normal alanine aminotransferase levels (Nw-N ALT), 7 individuals with normal-weight and elevated ALT levels (Nw-E ALT), 12 individuals with obesity and normal ALT levels and (Ob-N ALT), and 18 individuals with obesity and elevated ALT levels (Ob-E ALT). Fecal SCFA concentrations were quantified using gas chromatography, and gut microbiota was characterized by sequencing 16S rRNA. Median fecal SCFA concentrations (propionate, butyrate, and valerate) were highest in the Ob-E ALT group and lowest in the Nw-N ALT group (*p* < 0.05). These SCFA concentrations were also higher in obese than in normal-weight individuals regardless of ALT levels (*p* < 0.05) and in elevated ALT individuals regardless of obesity status, although the difference lost significance after correction for multiple testing. Notably, the abundance of *Roseburia* was higher in Ob-N ALT and Ob-E ALT than in the Nw-N ALT group and correlated positively with fecal SCFA concentrations. In conclusion, this pilot study suggests that the presence of both obesity and persistent elevated serum ALT levels is associated with increased fecal SCFA concentrations and SCFA-producing bacteria, particularly *Roseburia*. However, a larger sample is required to define whether the associations of SCFA fecal levels with obesity and elevated ALT levels are independent.

## 1. Introduction

Nonalcoholic fatty liver disease (NAFLD) is an increasing health concern with a worldwide prevalence of 25%–30% in adults, varying according to ethnicity and geographic region. In particular, the population of Latin America, including Mexico, has a higher prevalence of NAFLD and NAFLD-associated cardiometabolic risk factors [1, 2]. As a biomarker of liver function, serum alanine aminotransferase (ALT) levels are commonly used to evaluate hepatic inflammation and liver injury in chronic liver diseases, including NAFLD patients [3, 4]. While obesity is the most common NAFLD risk factor, normal-weight individuals can also develop NAFLD, although with a lower prevalence. It has been suggested that the pathophysiologic mechanisms of the disease are different in normal weight and obesity, which are not fully understood [5].

Metabolite imbalance caused by gut microbiota dysbiosis has also been considered an NAFLD risk factor. Short-chain fatty acids (SCFAs) such as acetate, propionate, and butyrate are gut microbiota fermentation products from undigested fiber and carbohydrates, found to be increased both in circulation and feces of individuals with NAFLD and hepatic fibrosis [6, 7]. However, some studies have reported decreased SCFA concentrations in NAFLD [8, 9]. These discrepancies could be related to several confounders such as obesity status, sex, diet, and gut microbiota [10], which should be considered when analyzing SCFA levels.

Although the increase of both SCFA concentrations and SCFA-producing bacteria has been associated with a better metabolic profile [11–13], other studies have suggested that SCFA overproduction may promote obesity by increased energy production [14–17]. Thus, associations of SCFA levels and gut microbiota profiles with hepatic steatosis may be confounded by the presence of obesity. Whether the SCFA imbalance is associated with hepatic steatosis in both normal-weight and obese individuals has not been clearly established. We thus conducted a pilot study to identify differences in SCFA fecal concentrations and SCFA-producing gut bacteria in individuals with normal serum ALT without hepatic steatosis and subjects with persistently high ALT and hepatic steatosis, with and without the influence of obesity. In addition, this pilot study was conducted to estimate the sample size needed for subsequent study in Mexican population.

## 2. Materials and Methods

### 2.1. Study Design

The study used a nested case–control design in The Health Workers Cohort Study (HWCS) in Mexico to compare fecal SCFA concentrations of 30 adult subjects with obesity: 18 with persistent elevated ALT levels (Ob-E ALT) and 12 with normal ALT levels (Ob-N ALT); and 14 normal-weight adult individuals: 7 with persistent elevated ALT levels (Nw-E ALT) and 7 with normal ALT levels (Nw-N ALT). All participants had to meet the following inclusion criteria: male or female adult individuals (> 18 years); liver ultrasound confirming that individuals with elevated ALT levels had hepatic steatosis, while those with normal ALT levels did not; no significant alcohol consumption, no competing etiologies for hepatic steatosis or coexisting causes of chronic liver disease, as previously described [18].

The study was approved by the Ethics Committees of INMEGEN (346-05/2018/I) and of the Mexican Social Security Institute (12CEI 09 006 14). All participants signed informed consent, and the study was conducted following the guidelines detailed in the Declaration of Helsinki.

Anthropometric measures were performed by standardized procedures, and blood samples were drawn after 8–12 h overnight fast for biochemical measurements, as previously described [19]. Individuals were grouped according to body mass index (BMI) as with normal-weight (BMI > 18.5 and < 25 kg/m^2^) or obesity (BMI ≥ 30 kg/m^2^). Type 2 diabetes (T2D) was defined considering a fasting glucose measurement ≥ 126 mg/dL or previous self-reported diagnosis [20]. Normal ALT serum levels were defined as < 40 U/L, while persistent elevated ALT levels were defined as ≥ 40 U/L in at least two independent measurements as previously described [18].

### 2.2. Habitual Dietary Intake Assessment

A semiquantitative food frequency questionnaire (FFQ) previously validated in the Mexican population was used to evaluate habitual dietary intake [21]. Daily consumed grams of proteins, carbohydrates, and lipids were converted to kilocalories using the corresponding Atwater factor. In this manner, the consumption of each macronutrient was expressed as the percentage of the daily energy intake. The total dietary fiber intake in grams was standardized per 1000 kilocalories to reduce interindividual energy intake variation.

### 2.3. Fecal SCFA Quantification

Fecal samples were collected in a sterile container, cold-chain-transported, and stored at −80°C until required for processing. Acetate, propionate, butyrate, valerate, isobutyrate, and isovalerate concentrations were measured from fecal waters obtained from 75 mg of feces. Hydrochloric acid was added to adjust the pH of the aqueous sample to 2.0. The samples were incubated to room temperature for 10 min, and the homogenate was centrifuged at 14,000 rpm for 10 min. One microliter of the supernatant aquos was injected in a splitless mode in a gas chromatograph (7820A model, Agilent Technologies, Santa Clara, CA, USA) equipped with a flame ionization detector. SCFAs were separated using a capillary column (30 m × 0.25 mm × 0.25 μm; J&W Agilent Technologies). Individual SCFAs were identified based on their retention times using referent standards for detecting these fatty acids (WSFA-2 Mix, Supelco, Darmstadt, Germany). Three outliers identified by Grubbs' test were excluded from the analyses (two from the Ob-E ALT group and one from the Nw-N ALT group). Fecal concentrations of SCFAs were expressed in mg/g wet weight of feces. In this report, the total SCFA concentration was defined as the sum of the individual SCFAs.

### 2.4. DNA Extraction and 16S rRNA Sequencing

DNA was extracted using a QIAamp DNA Stool (Qiagen, Hilden, Germany), with a previous step of mechanical sample lysis with a FastPrep device. DNA concentration and purity were determined by a spectrophotometry (Nanodrop 2000c, Thermo Scientific, Wilmington, DE, USA). The 16S rRNA gene V4 hypervariable region from 30 participants (5 Nw N-ALT, 6 Nw E-ALT, 8 Ob N-ALT, and 11 Ob E-ALT) was sequenced using the 515F and 806R primers as described elsewhere [19]. Libraries were sequenced on the Illumina MiSeq 2 × 250 platform (Illumina, San Diego, CA, USA) at the Sequencing Unit of the Instituto Nacional de Medicina Genómica.

### 2.5. Sequence Data Processing

Sequence data were processed in a similar manner to that described by López-Montoya et al. [22]. Briefly, the paired-end raw files were processed using the QIIME2 pipeline (Quantitative Insights Into Microbial Ecology 2) [23]. In order to remove adapters, sequences with barcode mismatches, and low-quality reads (Phred values < 30), DADA2 software (v1.20.0) was used. Chimeric sequences were removed using the “consensus” method, and reads were denoised to obtain amplicon sequence variants (ASVs) [24]. Taxonomic classification was assigned using the SILVA v138-99 reference database. The ASVs were aligned with the MAFFT algorithm. All artifacts and metadata files were imported in R using the qiime2R package (v0.99.34) to generate a phyloseq object. Thereafter, samples were standardized by rarefaction at a 37,000 high-quality read depth.

### 2.6. Bioinformatic Analysis

Data analyses were performed in the R environment (v4.2.3). Alpha diversity metrics (observed ASV and Shannon index) were estimated by the plot richness function. Weighted and unweighted UniFrac distances were used to estimate beta diversity. A permutational multivariate analysis of variance (PERMANOVA) was used to test differences in beta diversity among study groups using the Vegan package (v2.5.7), applying the adonis2 function with default parameters (999 permutations). Microbial composition differences among study groups from the phylum to genus levels were assessed by linear discriminant analysis effect size (LEfSe v1.0) [25]. LDA scores > 2.0 and *p* < 0.05 were considered statistically significant.

### 2.7. Statistical Analysis

Quantitative variables were compared using the Kruskal–Wallis and post hoc Dunn's multiple comparison tests or the Mann–Whitney U test. Categorical variables were compared using the chi-square test. These analyses were performed using the statistical package SPSS Statistics (IBM SPSS Statistics, version 24, Chicago, IL, USA). Spearman correlation coefficients between SCFA concentrations and biochemical and dietary measurements and between relative abundance of genera and SCFA concentrations were calculated. The resulting *p*-value was corrected for multiple comparisons with the false discovery rate method. The sample size was fixed by the available study population [18]. The sample size required for future studies was calculated based on the results of this pilot study to achieve 90% power and a significance level of 1% to detect differences in SCFA means between the study groups (Nw-N ALT vs. Nw-E ALT and Ob-N ALT vs. Ob-E ALT) [26].

## 3. Results

### 3.1. Fecal SCFAs in Hepatic Steatosis

The anthropometric and biochemical characteristics of the study groups are described in Table 1. Significant differences in serum ALT, AST, and glucose levels were observed among groups, being higher in subjects with persistent elevated serum transaminase and hepatic steatosis, regardless of the presence of obesity. Ob-E ALT subjects showed a lower median age than the other study groups, while the Ob-N ALT group showed the highest frequency of female participants (91.7%). There were no significant differences in total cholesterol or triglyceride serum levels among study groups. Moreover, no differences in total calorie and dietary fiber intake or macronutrient proportions as assessed by an FFQ were observed.

**Figure 1:**
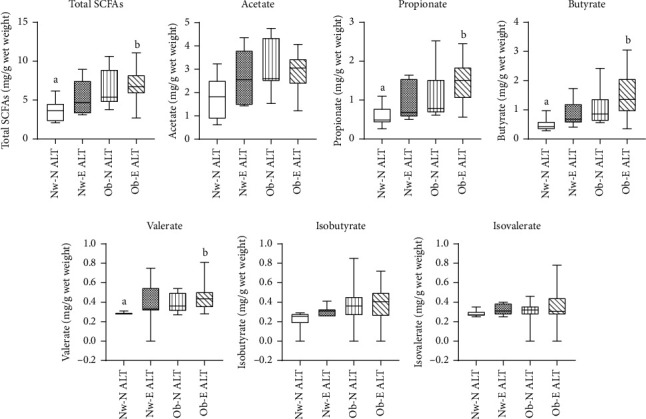
Comparison of fecal short-chain fatty acid concentrations across study groups. Data are presented in box plots with median, interquartile range, and bars represent minimum and maximum. Across groups, different superscript letters indicate significantly different medians using the Kruskal–Wallis test for independent samples and post hoc Dunn's multiple comparison test (*p* < 0.05). Three outliers were identified by Grubbs' test, which were excluded from the analyses. Nw-N ALT (normal-weight and normal ALT levels), Nw-E ALT (normal-weight and elevated ALT levels), Ob-N ALT (obesity and normal ALT levels), and Ob-E ALT (obesity and elevated ALT levels).

**Figure 2:**
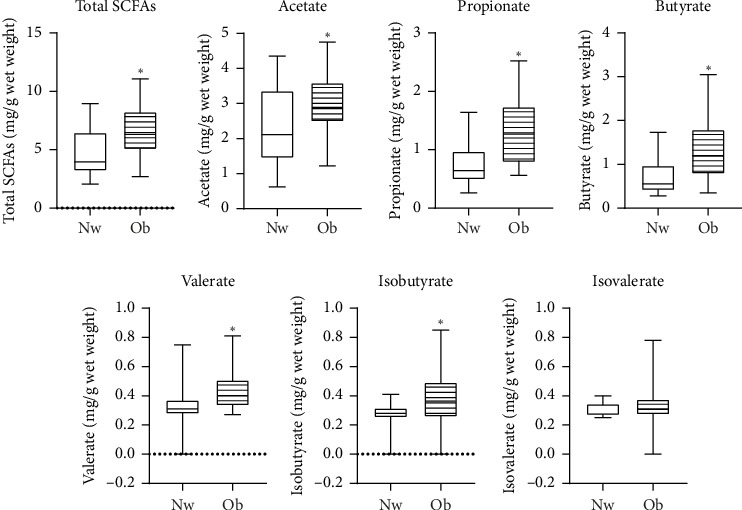
Comparison of short-chain fatty acid fecal concentrations between individuals with normal weight (*n* = 13) and obesity (*n* = 28). Data are presented in box plots with median and interquartile range, and bars represent minimum and maximum. An asterisk indicates significantly different medians using the Mann–Whitney U test (*p* < 0.05). Nw (normal weight) and Ob (obesity).

Figure 1 compares fecal SCFA concentrations among the four study groups. Total and individual fecal SCFA concentrations (propionate, butyrate, and valerate) were highest in Ob-E ALT and lowest in Nw-N ALT individuals, showing statistically significant differences after adjusting for multiple comparisons (*p* < 0.05). Individuals with obesity (regardless of ALT levels and hepatic steatosis) had significantly higher total and acetate, propionate, butyrate, valerate, and isobutyrate concentrations as compared to normal-weight individuals (*p* < 0.05 after adjusting for multiple comparisons; Figure 2). Propionate, butyrate, and valerate levels were also higher in individuals with elevated ALT regardless of obesity status, although the difference lost significance after correction for multiple testing.

### 3.2. Fecal SCFA Correlations With Metabolic and Dietary Parameters

**Figure 3:**
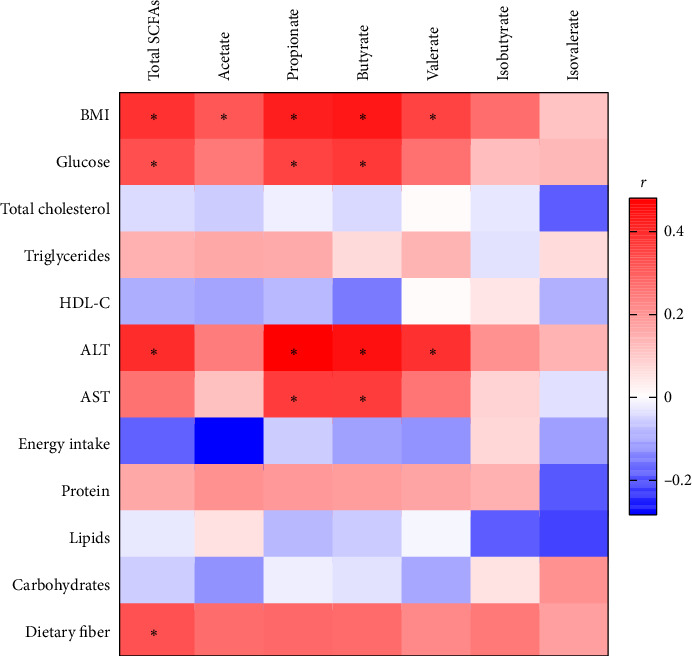
Heatmap correlation of short-chain fatty acid fecal levels with anthropometric, biochemical, and dietary macronutrient parameters. The color scale indicates Spearman's correlation coefficients. ^∗^Indicates a significant correlation < 0.05.

As expected, total SCFA, acetate, propionate, butyrate, and valerate fecal concentrations positively correlated with BMI (*p* < 0.05), and all except acetate correlated positively and significantly with ALT levels. Notably, although no dietary macronutrient (protein, lipid, and carbohydrate) correlated significantly with individual or total fecal SCFAs, dietary fiber intake showed significantly positive correlations with total SCFA (rho = 0.327; *p*=0.037) and borderline significant correlations with acetate (rho = 0.276; *p*=0.08), propionate (rho = 0.283; *p*=0.073), and butyrate (rho = 0.277; *p*=0.08). Total SCFA, propionate, and butyrate concentrations also correlated positively with serum glucose levels (rho = 0.330, 0.353, and 0.373, *p* < 0.05, respectively; Figure 3). However, all correlations lost significance after correction for multiple testing.

### 3.3. SCFA Differences According to Sex and T2D Status

No significant fecal SCFA concentration differences were observed according to sex or T2D status (Supporting Tables 1 and 2). Because the proportion of women in the Ob-N ALT group was 91.7%, we compared fecal SCFA levels only among women without T2D in the four study groups. Total SCFA, acetate, propionate, and butyrate concentrations were significantly higher in Ob-E ALT than in Nw-N ALT women, after adjusting for multiple comparisons (*p* < 0.05; Supporting Figure 1).

### 3.4. Gut Microbiota in Hepatic Steatosis

Both alpha diversity indices (observed ASV and Shannon) were reduced in the Nw-E ALT, Ob-N ALT, and Ob-E ALT groups as compared to those in Nw-N ALT controls, but only the difference between Nw-N ALT and Ob-E ALT was significant after adjusting for multiple comparisons (*p* < 0.05; Supporting Figure 2). Similarly, unweighted UniFrac-based beta-diversity analyses showed significant differences only between Ob-E ALT and Nw-N ALT controls (Supporting Table 3).

**Figure 4:**
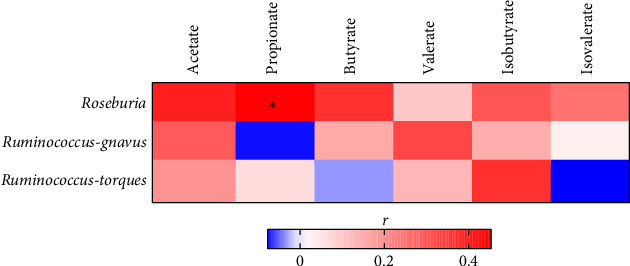
Heatmap showing the correlations among abundances of genera increased in Ob-N ALT/Ob-E ALT and fecal short-chain fatty acid concentrations. The color scale indicates Spearman's correlation coefficients. ^∗^Indicates a significant correlation < 0.05.

Ob-N ALT individuals showed a significantly increased abundance of *Roseburia*, *Ruminococcus-gnavus,* and *Ruminococcus-torques* genera, while the Ob-E ALT group showed a significantly higher abundance of only *Roseburia* as compared to the Nw-N ALT group (Supporting Figure 3). No significant differences in bacterial abundance were observed in elevated ALT as compared to those in normal ALT groups (Nw-E ALT vs. Nw-N ALT and Ob-E ALT vs. Ob-N ALT). We thus tested the correlation of the abundance of these three bacteria with fecal SCFAs in the entire sample. Notably, only *Roseburia* (more abundant in Ob-N ALT and Ob-E ALT) showed a positive and significant correlation with fecal propionate (*p*=0.02) and a similar trend for acetate (*p*=0.05) and butyrate (*p*=0.07, Figure 4). However, these correlations lost significance after correction for multiple testing.

## 4. Discussion

Although the metabolic effects of SCFAs have been widely described, their roles in obesity and hepatic liver disease remain controversial. Some studies have suggested that SCFA overproduction may promote obesity by increasing energy generation [14–17], while others suggest it is associated with lower body weight and metabolic benefits [11–13]. These inconsistencies could be partly due to different environmental, demographic, and genetic factors related to the study populations [10, 27]. In the current study, subjects with obesity regardless of transaminase levels showed higher fecal SCFA concentrations, consistent with various previous studies [17, 28, 29].

Interestingly, individuals with both obesity and elevated ALT levels had the highest acetate, propionate, butyrate, and valerate fecal concentrations, although differences were significant only when compared to the NW individuals without elevated ALT levels. This is consistent with observations of previous studies comparing fecal SCFA concentrations in subjects with NAFLD and healthy controls [6, 7]. Moreover, increased SCFAs, mainly propionate, have been causally associated with other metabolic traits such as T2D [30], and propionate supplementation increased the risk of insulin resistance and weight gain [31]. We did not observe significant differences in fecal SCFA concentrations in patients with T2D, most likely because of the small sample size. Although fiber intake was not associated with obesity regardless of ALT levels, we observed a positive correlation of total SCFA fecal concentrations and fiber intake, which is consistent with various studies that consider dietary fiber as the main substrate for SCFA production by gut microbiota [32].

Even though in the present study SCFA concentrations were measured in feces and not in circulation, Thing et al. reported higher propionate and valerate plasma concentrations in patients with hepatic steatosis, and plasma butyrate concentrations were significantly associated with fibrosis severity [33]. However, these findings are inconsistent with several reports demonstrating metabolic health benefits of certain SCFAs, such as butyrate and propionate [11–13]. In this regard, SCFA concentrations excreted in feces result from both microbiota SCFA production and host uptake. SCFAs are transported to the liver through the portal circulation, where propionate and butyrate may be used as an energy source by hepatocytes. Consequently, only a fraction of SCFAs reaches the systemic circulation [34]. Thus, future studies measuring SCFAs in feces, portal and systemic circulation, could help clarify these inconsistencies.

Several studies have shown that SCFAs are involved in the regulation of pathways implicated in hepatic steatosis and inflammation through the gut–liver axis, with effects that are not always beneficial. Elevated SCFA concentrations can contribute to metabolic diseases by activating G-protein-coupled receptors, which stimulates the peptide–YY secretion that inhibits gut motility. This may increase the absorption of SCFAs and other nutrients leading to hepatic lipid accumulation [35, 36]. Moreover, Rau et al. [6] found that fecal concentrations of propionate and acetate correlated positively with the inflammation-related Th17/Treg ratio in peripheral blood, which also increased in NAFLD patients. In addition, in a TLR5-KO mouse model, SCFAs and mild chronic inflammation promoted increased hepatic lipogenesis and metabolic syndrome [37], while increased SCFA production in response to soluble fiber supplementation (inulin) conferred a higher risk of hepatocellular carcinoma [38] and dietary fructose enhanced hepatic lipogenesis through microbiota-derived acetate in a murine model [39]. Altogether, these findings suggest that whether SCFAs are beneficial or harmful to the host is highly context-dependent.

It has been suggested that increased fecal and circulating SCFA levels in individuals with obesity and NAFLD result from a higher abundance of SCFA-producing bacteria [6, 30]. Likewise, high fecal abundance of propionate and butyrate-associated bacterial enzymes were found in NAFLD patients with advanced fibrosis [40]. In our study, Ob-E ALT, Ob-N ALT, and Nw-E ALT individuals showed a lower gut microbiota alpha diversity than the Nw-N ALT control group, a sign of dysbiosis commonly observed in subjects with obesity and NAFLD [41, 42]. Notably, an increased abundance of *Roseburia*, *Ruminococcus-gnavus group, and Ruminococcus-torques group*, all known SCFA-producing bacteria [32, 43], was observed in subjects with obesity and normal ALT levels, but only *Roseburia* was increased in the Ob-E ALT group. Although *Roseburia* has been associated with metabolic benefits [44], it is noticeable that the abundance of this genus has been consistently found to be higher in Mexican adults and children with obesity, metabolic syndrome, or diabetes [19, 45–50]. In our study, *Roseburia* abundance correlated with higher propionate fecal concentrations. Although experimental and genomic evidence show that butyrate is the major fermentation product of *Roseburia*, certain species have genes capable of producing acetate and propionate [50]. Therefore, metagenomic studies are needed to identify *Roseburia* species associated with obesity and related metabolic diseases.

Some limitations of the study should be acknowledged. First, although hepatic ultrasound studies confirmed the presence or absence of hepatic steatosis, we could not establish the presence of inflammation or fibrosis. Because SFCA fecal concentrations are high in patients with hepatic fibrosis, this could be a confounding factor in our study [40]. In addition, although we used an FFQ validated in the Mexican population to assess habitual diet, it is known that this instrument may overestimate the intake of some nutrients, including fiber [51]. Moreover, although we assessed some factors known to modify SCFA fecal concentrations such as diet and abundance of SCFA-producing bacteria, other factors such as intestinal transit time and/or SCFA consumption by the bacterial community were not evaluated [52, 53]. Finally, the small sample size of this pilot study likely explains the lack of association of SCFA levels with T2D status and sex and the inability to define whether the associations of SCFA fecal levels with obesity and elevated ALT levels are independent. Based on our results, to achieve 90% statistical power, a sample size of 51 per group is required to identify fecal propionate, butyrate, and acetate level differences between Nw-N ALT and Nw-E ALT groups, while a sample size of 107 is required to identify differences in fecal propionate and butyrate levels between Ob-N ALT and Ob E-ALT groups. A total of 1260 subjects per group would be required to identify significant differences in fecal acetate levels between Ob N-ALT and Ob E-ALT groups.

## 5. Conclusions

Our pilot study suggests that the presence of both obesity and persistent elevated serum ALT levels is associated with increased fecal SCFA levels and the abundance of SCFA-producing bacteria, particularly *Roseburia*. In addition, although increased fecal SCFA concentrations were associated with higher dietary fiber intake and *Roseburia* abundance, future studies in larger samples, measuring fecal and circulating SCFA concentrations, are required to clarify SCFA role in metabolic diseases.

## Figures and Tables

**Table 1:** Comparison of anthropometric, biochemical, and dietary macronutrient parameters among study groups.

	Nw-N ALT (*n* = 7)	Nw-E ALT (*n* = 7)	Ob-N ALT (*n* = 12)	Ob-E ALT (*n* = 18)	*p-*value
Age (years)	66.0 (62.0–68.0)^a,b^	64.0 (57.0–74.0)^a,b^	70.0 (58.3–72.8)^a^	57.0 (43.8–64.8)^b^	0.035
Female *n* (%)	5 (71.4%)	3 (42.9%)	11 (91.7%)	10 (55.6%)	0.104
BMI (kg/m^2^)	23.2 (22.0–24.5)^a^	24.0 (23.6–24.5)^a^	33.3 (31.2–35.8)^b^	32.5 (30.7–35.7)^b^	< 0.001
Diabetes *n* (%)	1 (14.3)	1 (14.3)	3 (25.0)	6 (33.3)	0.677
Glucose (mg/dL)	98.0 (93.0–100.0)	94.0 (93.0–101.0)	102.5 (96.5–106.0)	108.0 (100.3–132.3)	0.047
Total cholesterol (mg/dL)	210.0 (175.0–224.0)	195.0 (161.0–224.0)	180.5 (157.3–227.8)	202.0 (159.3–239.8)	0.615
Triglycerides (mg/dL)	141.0 (120.0–154.0)	125.0 (94.0–202.0)	178.5 (134.8–192.5)	161.0 (108.0–182.5)	0.666
LDL-C (mg/dL)	119.0 (92.6–139.9)	88.6 (80.3–113.4)	96.5 (83.9–140.1)	120.6 (85.6–151.3)	0.397
HDL-C (mg/dL)	59.1 (43.5–67.0)	41.7 (36.0–66.8)	45.4 (35.0–56.2)	48.9 (42.0–57.5)	0.312
ALT (U/L)	20.0 (16.0–22.0)^a,b^	47.0 (42.0–53.0)^b,c^	20.0 (15.0–25.3)^a^	70.5 (46.8–116.3)^c^	< 0.001
AST (U/L)	22.0 (19.0–27.0)^a^	36.0 (29.0–38.0)^a,b^	22.0 (20.3–25.5)^a^	54.0 (41.3–75.5)^b^	< 0.001
Total energy intake (kcal/day)	998.5 (845.5–2211.2)	1032.3 (685.8–1554.3)	1323.8 (770.0–1750.9)	1572.5 (989.4–1936.1)	0.390
Proteins (%)	11.3 (8.0–13.7)	14.4 (10.1–18.1)	14.3 (11.9–15.1)	13.7 (12.2–15.9)	0.073
Lipids (%)	21.6 (16.6–24.1)	26.7 (23.3–30.0)	21.2 (17.0–26.2)	21.0 (18.9–29.6)	0.303
Carbohydrates (%)	67.0 (62.5–73.8)	60.5 (50.7–65.3)	64.3 (59.2–68.1)	66.0 (55.1–68.9)	0.190
Dietary fiber (g/1000 kcal)	8.1 (7.3–9.0)	6.7 (6.3–9.9)	8.3 (7.0–11.9)	8.7 (7.6–10.4)	0.518

*Note:* Data are presented as median (interquartile range) or numbers (percentages). Data were analyzed using Kruskal–Wallis and post hoc Dunn's multiple comparison tests or chi-square test. Across a row, different superscript letters indicate significantly different values after adjusting for multiple comparisons (*p* < 0.05). The lack of superscript letters indicates no significant differences among groups. ALT (alanine aminotransferase), AST (aspartate aminotransferase), Nw-N ALT (normal-weight and normal ALT levels), Nw-E ALT (normal-weight and elevated ALT levels), Ob-N ALT (obesity and normal ALT levels), and Ob-E ALT (obesity and elevated ALT levels).

Abbreviations: BMI, body mass index; HDL-C, high-density lipoprotein cholesterol; LDL-C, low-density lipoprotein cholesterol.

## Data Availability

Data supporting this study are available on reasonable request.

## Supporting Information

Supporting Figure 1: Comparison of the fecal short-chain fatty acid concentrations across study groups including only females without type 2 diabetes.

Supporting Figure 2. Comparison of alpha diversity among groups.

Supporting Figure 3. LEfSe plots showing differentially abundant genera.

Supporting Table 1: Fecal short-chain fatty acid concentrations stratified by sex.

Supporting Table 2: Fecal short-chain fatty acid concentrations stratified by type 2 diabetes status.

Supporting Table 3. Unweighted UniFrac-based beta-diversity analyses.
