# Supplementary material for: Association of Gut Microbiota-Derived Short-Chain Fatty Acids With Persistent Elevated Serum Transaminase Levels in Normal Weight and Obesity: A Pilot Study
Source: J Nutr Metab. 2025 Sep 20;2025:6652392. doi: 10.1155/jnme/6652392 (PMC12476282; doi:10.1155/jnme/6652392)
Supplement: Supporting Information — Additional supporting information can be found online in the Supporting Information section. [file 6652392.f1.docx]

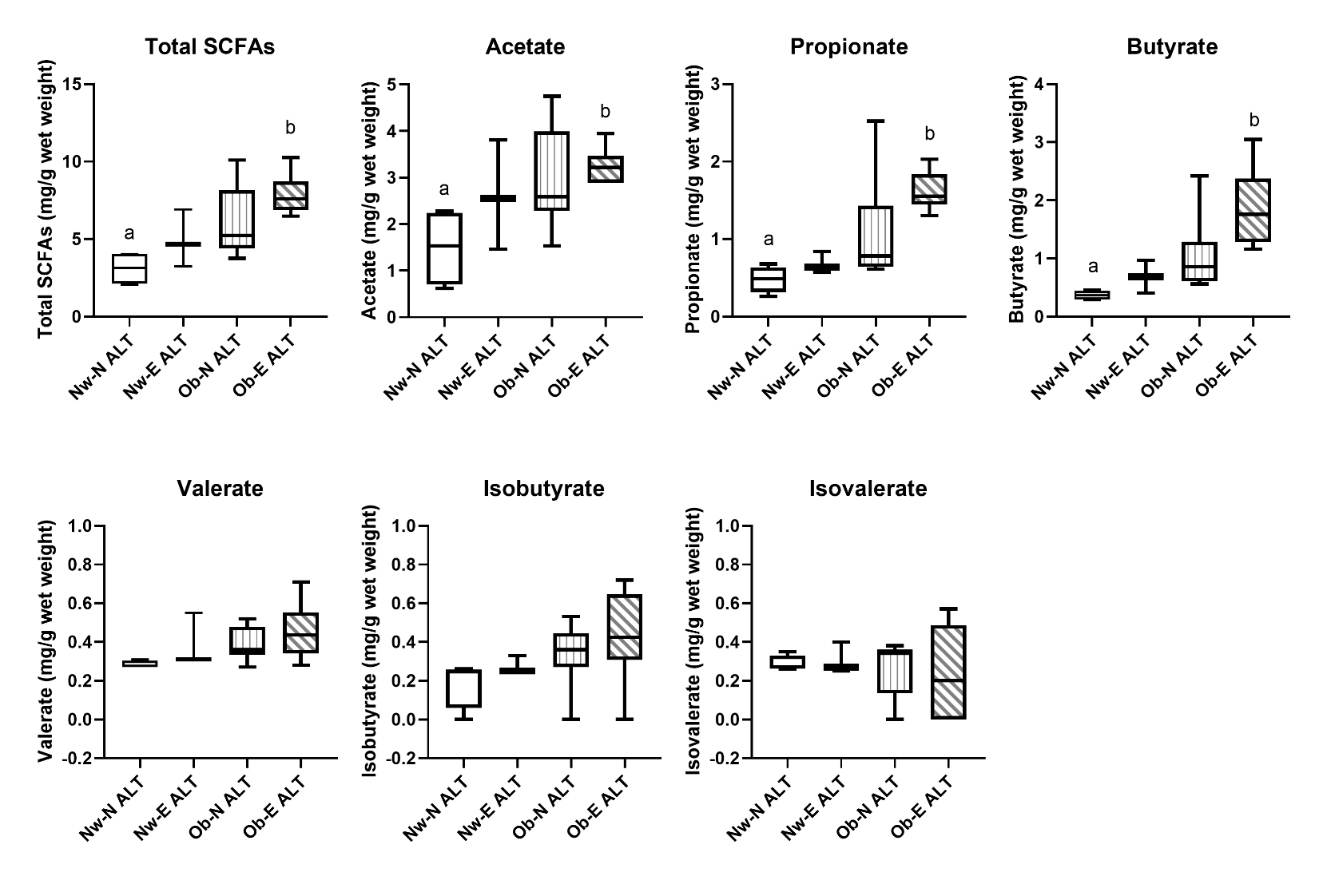


**Supplementary Figure 1.** Comparison of the fecal short-chain fatty acids concentrations across study groups including only females without type 2 diabetes. Data are presented in box plots with median and interquartile range, and bars indicate minimum and maximum. Across groups, different superscript letters indicate significantly different medians using the Kruskal-Wallis test for independent samples with Dunn’s post hoc test (*P*<0.05). Three outliers were identified by Grubbs’ test, which were excluded from the analyses. Nw-N ALT (normal-weight and normal ALT levels), Nw-E ALT (normal-weight and elevated ALT levels), Ob-N ALT (obesity and normal ALT levels) and Ob-E ALT (obesity and elevated ALT levels).


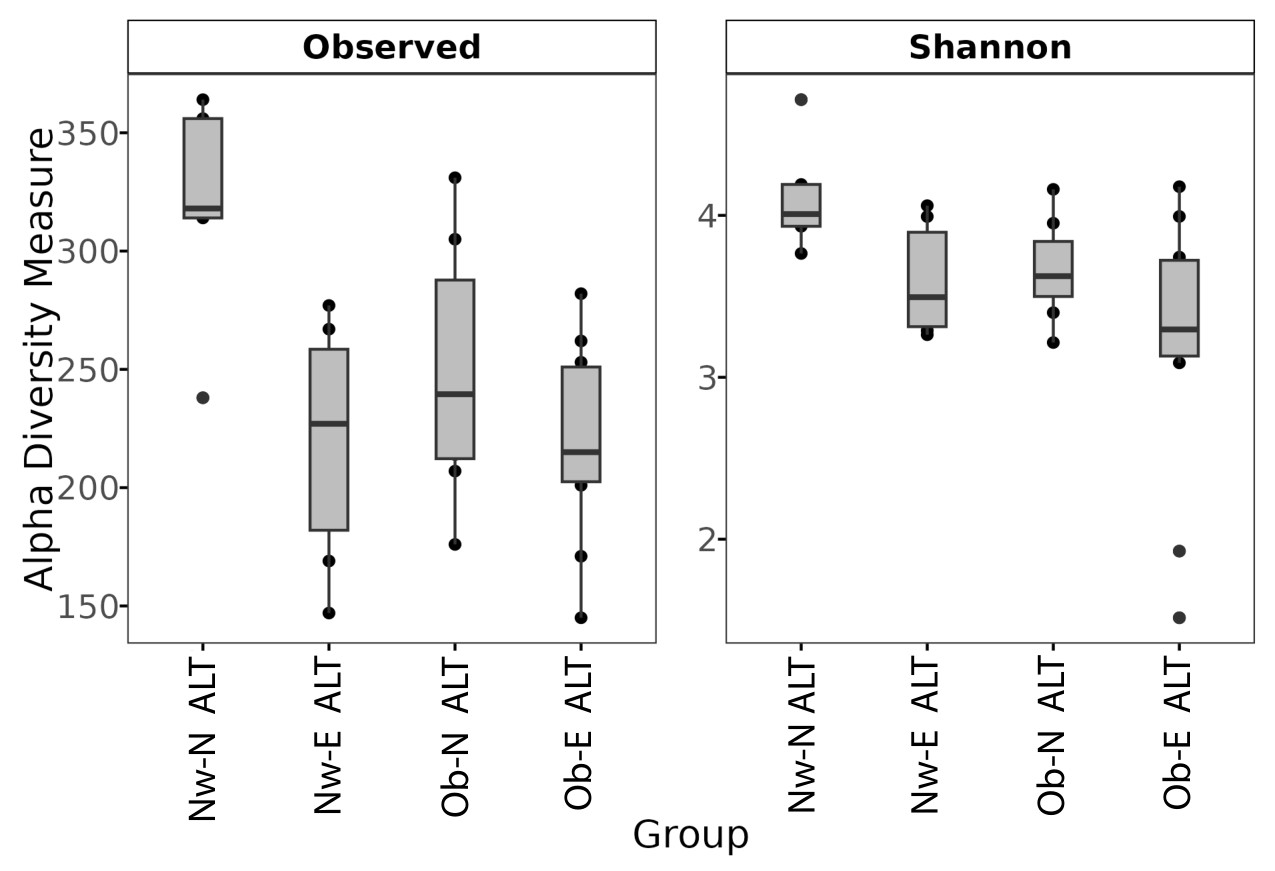


**Supplementary Figure 2.** Comparison of alpha diversity among groups. The plotted data represent medians and interquartile ranges. Observed ASV and Shannon indices were significant between Nw-N ALT and Ob-E ALT after adjusting for multiple comparisons (*P*<0.05). Nw-N ALT (normal-weight and normal ALT levels), Nw-E ALT (normal-weight and elevated ALT levels), Ob-N ALT (obesity and normal ALT levels) and Ob-E ALT (obesity and elevated ALT levels).


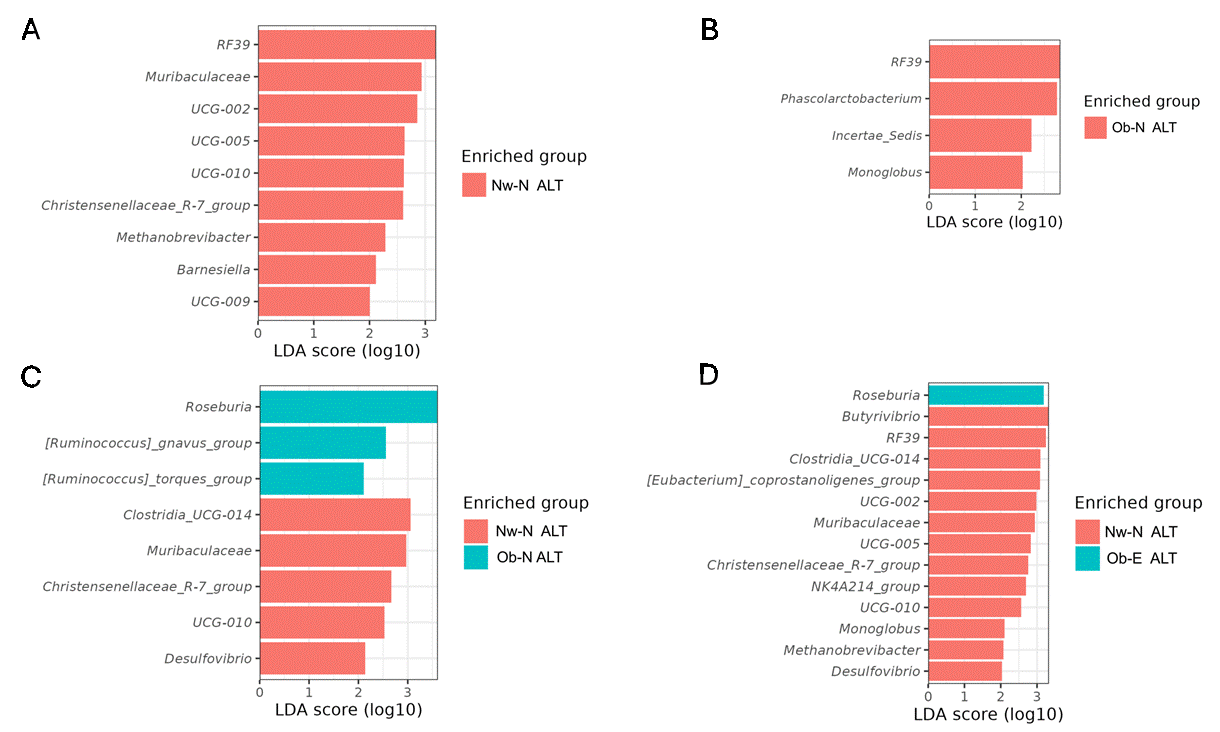


**Supplementary Figure 3.** LEfSe plots showing differentially abundant genera between: (A) Nw-N ALT and Nw-E ALT group; (B) Ob-N ALT and OB-E ALT group; (C) Nw-N ALT and Ob-N ALT group and (D) Nw-N ALT and Ob-E ALT group. Only genera with LDA score > 2.0 and *P* < 0.05 are shown. Nw-N ALT (normal-weight and normal ALT levels), Nw-E ALT (normal-weight and elevated ALT levels), Ob-N ALT (obesity and normal ALT levels) and Ob-E ALT (obesity and elevated ALT levels).

| **Supplementary Table 1. Fecal short chain fatty acid concentrations stratified by sex.** | | | | |
| --- | --- | --- | --- | --- |
|  | **Female**  (n=26) | **Male**  (n=15) | ***P*-value** |  |
| **Total SCFAs** (mg/g wet weight) | 5.37(4.13-7.47) | 6.17(3.77-8.24) | 0.725 |  |
| **Acetate** (mg/g wet weight) | 2.59(2.10-3.28) | 3.23(1.53-3.63) | 0.552 |  |
| **Propionate** (mg/g wet weight) | 1.01(0.63-1.54) | 0.99(0.67-1.63) | 0.705 |  |
| **Butyrate** (mg/g wet weight) | 0.91(0.59-1.52) | 0.97(0.61-1.40) | 0.685 |  |
| **Valerate** (mg/g wet weight) | 0.35(0.30-0.46) | 0.38(0.29-0.51) | 0.725 |  |
| **Isobutyrate** (mg/g wet weight) | 0.34(0.24-0.42) | 0.30(0.24-0.40) | 0.675 |  |
| **Isovalerate** (mg/g wet weight) | 0.28(0.25-0.36) | 0.28(0.27-0.36) | 0.551 |  |
| Data are medians (interquartile range). *P*-values were calculated with U de Mann-Whitney non-parametric tests. | | | | |

| **Supplementary Table 2. Fecal short-chain fatty acid concentrations stratified by type 2 diabetes status.** | | | | | |
| --- | --- | --- | --- | --- | --- |
|  | **Non-T2D**  (n=30) | **T2D**  (n=11) | ***P*-value** | |  |
| **Total SCFAs** (mg/g wet weight) | 5.37(3.92-7.59) | 6.02(5.17-8.95) | 0.301 | |  |
| **Acetate** (mg/g wet weight) | 2.59(1.95-3.35) | 2.70(2.29-4.07) | 0.578 | |  |
| **Propionate** (mg/g wet weight) | 0.81(0.61-1.50) | 1.18(0.79-1.55) | 0.650 | |  |
| **Butyrate** (mg/g wet weight) | 0.91(0.56-1.42) | 1.20(0.79-1.72) | 0.507 | |  |
| **Valerate** (mg/g wet weight) | 0.35(0.29-0.44) | 0.36(0.29-0.54) | 0.524 | |  |
| **Isobutyrate** (mg/g wet weight) | 0.32(0.24-0.42) | 0.30(0.24-0.40) | 0.674 | |  |
| **Isovalerate** (mg/g wet weight) | 0.29(0.26-0.36) | 0.28(0.26-0.31) | 0.735 | |  |
| Data are medians (interquartile range). *P*-values were calculated with U de Mann-Whitney non-parametric tests. (T2D) Type 2 diabetes. | | | |  |  |

| **Supplementary Table 3. Unweighted UniFrac-based beta-diversity analyses.** | | | | | |
| --- | --- | --- | --- | --- | --- |
|  | **F. Model** | **R^2^** | ***P*-value** | |  |
| Nw-N ALT vs Nw-E ALT | 1.403 | 0.135 | 0.064 | |  |
| Nw-N ALT vs Ob-N ALT | 1.294 | 0.105 | 0.060 | |  |
| Nw-N ALT vs Ob-E ALT | 1.532 | 0.099 | 0.030 | |  |
| Nw-E ALT vs Ob-N ALT | 1.168 | 0.089 | 0.179 | |  |
| Nw-E ALT vs Ob-E ALT | 1.018 | 0.064 | 0.512 | |  |
| Ob-N ALT vs Ob E ALT | 0.953 | 0.053 | 0.649 | |  |
| Nw-N ALT (normal-weight and normal ALT levels), Nw-E ALT (normal-weight and elevated ALT levels), Ob-N ALT (obesity and normal ALT levels) and Ob-E ALT (obesity and elevated ALT levels). | | | |  |  |
